# Mind-Body Therapies for African-American Women at Risk for Cardiometabolic Disease: A Systematic Review

**DOI:** 10.1155/2018/5123217

**Published:** 2018-02-26

**Authors:** Candace C. Johnson, Karen M. Sheffield, Roy E. Brown

**Affiliations:** ^1^Department of Family and Community Health Nursing, Virginia Commonwealth University School of Nursing, 1100 E. Leigh St., P.O. Box 980567, Richmond, VA 23298, USA; ^2^University of North Carolina Chapel Hill School of Nursing, 307 E. Carrington Hall, Campus Box 7460, Chapel Hill, NC 27599-7460, USA; ^3^Tompkins-McCaw Library for the Health Sciences, Virginia Commonwealth University School of Nursing, 509 N. 12th Street, P.O. Box 980582, Richmond, VA 23298, USA

## Abstract

**Background:**

A major determinant in cardiometabolic health is metabolic syndrome (MetS), a cluster of symptoms that portend the development of cardiovascular disease (CVD). As mind-body therapies are thought to help in lowering physiological and environmental CVD risk factors including blood pressure and psychological stress, they may also be beneficial for the primary prevention of CVD.

**Objectives:**

To synthesize and summarize existing knowledge on the effectiveness of mind-body therapies on MetS outcomes in African-American (AA) women, a US subpopulation at high risk for CVD.

**Search Methods:**

A systematic search of eight databases was conducted in order to identify published papers addressing the topic. We included trials involving AA adult women, ages 18–64, and we included RCTs that involved multifactorial interventions. Outcomes of interest were MetS, chronic disease, and CVD risk factors (blood pressure, blood lipids, blood glucose, BMI, waist circumference, and mental health domains). Two authors independently selected trials for inclusion, extracted data, and assessed risks of bias.

**Main Results:**

We identified five trials for inclusion in this review. One study reported outcomes associated with the full MetS symptom cluster. The included trials were small, short term, and at high risk of bias. All interventions lasted at least 6 weeks.

## 1. Introduction

The metabolic syndrome (MetS), which affects one-third [1] of the US population, is a constellation of clinical indicators characterized by multiple, interrelated metabolic abnormalities linked to insulin resistance. MetS is characterized by three of the following five cardinal clinical manifestations: fasting blood glucose (FBG) ≥ 100 mg/dL; waist circumference (WC) ≥ 88 cm; systolic blood pressure ≥ 130 mmHg and diastolic blood pressure ≥ 85 mmHg; high density lipoprotein (HDL) ≤ 39 mg/dL; and triglycerides ≥ 150 mg/dL [2]. National Health and Nutrition Examination Survey (NHANES) data reveal that the age-adjusted prevalence of MetS in African-American (AA) females (41.1%) rivals that of males of all racial/ethnic subgroups [3]. Additionally, worldwide, obesity, hypertension, and prediabetes rates [4] are highest in AA women when compared to all other racial/ethnic subgroups. Eighty-two percent of AA women are overweight and 59% obese compared to 74% and 41% of Hispanic women and 61% and 33% of White women, respectively [5].

Obesity is linked to inactivity and though physical activity (PA) is known to impact MetS [6, 7], AA women are the least active (36.1%) when compared to than their White (49.6%) or Hispanic (40.5%) counterparts and the overall population of US women (46.6%) [8]. AA women may lack knowledge about exercise recommendations, lack support to engage in exercise, or may face substantial barriers to engaging in exercise [9].

MetS precedes type 2 diabetes (T2D) and cardiovascular disease (CVD). The prevalence of having ≥2 risk factors for CVD is highest among AAs (48.7%) [10] and AA women experience higher age-adjusted blood pressure (BP) and death from CVD than all other ethnicities [11]. Among US ethnic and gender groups, AA women have the highest fasting glucose levels and more AAs are diagnosed with T2D at a rate 1.5 times that of Whites (21.3% versus 14%, resp.) [12]. Sixty-eight percent of T2D-related deaths occur in individuals who also had a CVD-related comorbidity; AA women have the highest rate of death from heart disease and complications from T2D [11]. Therefore, AA women are at highest risk for MetS compared with any other US subpopulation underscoring the urgent need for effective health promotion interventions.

In AAs, MetS is directly linked to central and overall obesity [6]. Standard treatment for obesity and the MetS is focused on diet and physical activity, though psychosocial stress has emerged as a factor that extends beyond the concept of energy imbalance [13]. In AA women, higher stress and other heritable—genetic and environmental—factors including lower education, older age, lower PA, higher BMI, and current smoking were associated with having MetS [14]. In AAs, low social status in the community, low social support, and the experience of race-based discrimination are associated with stress, depression, and mental health-related quality of life [15]. Likewise, many AA women identify with the Strong Black Woman's Syndrome [16, 17], an identity associated with sustained chronic stress states and depressive symptoms [18], which further increases risk for MetS [19].

The MetS symptom cluster leads to the pathogenesis of CVD via bidirectional neuroendocrine and central nervous system mechanisms. Possessing more than 2 components of MetS signifies higher risk than is predicted by its components when analyzed individually and adding more components further concentrates CVD risk. A MetS diagnosis can indicate an adjusted relative risk of CVD outcomes, which is approximately 2-fold [20]. Mounting evidence for chronic stress and negative mood states has demonstrated bidirectional relationships with insulin resistance and other components of the MetS. Mood disorders have been described as a multisystem syndrome reflecting an imbalance between adaptive and maladaptive mechanisms. Stress-response mechanisms are altered by environmental experiences, particularly early in life influencing fundamental, overlapping mechanisms, for example, glucose-insulin homeostasis, inflammatory processes, oxidative stress, and glucocorticoid signaling [21]. Likewise, prospective studies have shown that depression, a common comorbidity with MetS, increases females risk for developing T2D by 2-3-fold [22]. Sleep disturbances and fatigue are additional risk factors in AA women [23] and can lead to impaired physical and social functioning [24, 25].

## 2. Mind-Body Therapies

Mind-body modalities are a diverse group of healthcare practices that represent adjuncts to conventional care of MetS [26]. Use of mind-body practices has increased in the US in recent years. Approximately 30% of the US adult population engages in some form of mind-body practice (e.g., yoga, tai chi, qi gong, deep breathing, and meditation) with deep breathing being the most popular [27]. Mind-body modalities that use spinal manipulation such as yoga have demonstrated efficacy in improving lipid profiles and blood pressure. Additionally, yoga improves insulin sensitivity and is generally effective in reducing the risk of T2D [28, 29]. A comprehensive review found that yoga improved specific metabolic risk factors including glucose tolerance, anthropometric characteristics, oxidative stress, and sympathetic activation [30].

Growing evidence suggests that tai chi and qi gong, also considered spinal manipulation therapies, may improve aerobic capacity and reduce stress in as few as one session [36] and though lower engagement is found among AA women when compared to other US subgroups, the proportion of use of mind-body therapies among minorities has increased significantly [37]. While research is limited, findings suggest the use of relaxation practices such as deep breathing programs reduces stress and anxiety in disadvantaged populations, including AA women [38]. Randomized controlled trials of transcendental and mindfulness meditation, a mind-body modality that elicits the parasympathetic (relaxation) response, have demonstrated favorable effects on systolic and diastolic blood pressure [39]. Fifteen AA adults experienced with mindfulness meditation training would recommend it but suggested that its presentation be culturally enhanced including familiar spiritual ideology and cultural practices [40].

Although the mechanisms underlying the psychological and biological effects of MetS are not well understood, the manifestations of the symptom cluster likely occur through multiple bidirectional autonomic and neuroendocrine pathways. Mind-body therapies may reduce the effects of both* psychological* (reflecting objective demands in relation to one's coping resources) and* systemic* stress (caused by physiological challenges such as injury, infection, or inflammation) [41]. The demands of modern society may be responsible for higher levels of chronic stress, leading to activation of the neurohormonal system, specifically the sympathoadrenal system and HPA axis that involves catecholamine release, vagal withdrawal, and cortisol secretion [42]. While research remains limited, findings suggest that the use of mind-body practices reduces the accumulated impact of psychological and systemic stress, inflammatory processes, and neurodegeneration. Mind-body practices also improve associated health outcomes such as feelings of well-being and multiple positive effects on autonomic and cognitive function [41]. These benefits may be particularly important for people at risk for the MetS and who may be more vulnerable to compromised immune systems—conditions for which AA women often experience disparate health outcomes [38].

The objective of this paper is to synthesize and summarize existing knowledge on the effectiveness of mind-body therapies on MetS outcomes in AA women. This systematic review critically examines the research of mind-body therapies as complementary therapies for management of the MetS focusing on single components and/or the aggregate of symptoms that make up the MetS rather than solely focusing on studies that have investigated all 5 components of the MetS.

## 3. Methods

A systematic search of eight databases was conducted along with a search of the reference list of the retrieved publications, in order to identify published papers addressing the topic. The databases searched were PubMed/Medline, CINAHL (EBSCO), Web of Science, Dissertations & Theses (ProQuest), Sociological Abstracts (ProQuest), Academic Search Complete (EBSCO), AMED (the Allied and Complementary Medicine Database) (EBSCO), and the Cochrane Library. The articles included were limited to those published from 2000 to 2016 that were written in English and involved adult aged female participants of ages 18–64 years. Medical Subject Headings (MeSH) and equivalent controlled vocabulary and keywords were utilized in each database as appropriate. The search was broken into two concept groups. One group encompassed the terminology used to describe “African Americans”; the other covered the terms relevant to “yoga” and “tai chi.” The last search was conducted on December 1, 2016.

### 3.1. Study Selections

For the purposes of this systematic review, mind-body therapies were limited to yoga, tai chi, and qigong; breathing exercises; mindfulness-based techniques; and any form of meditation. Studies that assessed one of the defined mind-body therapies alone or as an adjuvant to conventional treatment in human subjects with the MetS were included. Trials were excluded if the study was aimed at the development of methodology of mind-body therapy procedures without clinical outcomes, reported no data or statistical comparisons, or assessed healthy subjects only. Trials were included if the study examined a mind-body modality as part of a complex intervention (i.e., combining a mind-body therapy with other complementary modalities) or if the study was limited to only components of the MetS (i.e., hypertension or insulin sensitivity alone). Studies before 2000, abstracts, qualitative study designs, and those involving children, males only, pregnant women, individuals with a history of psychosis, and older adults were excluded. Dissertations/theses were initially screened for relevance but excluded in the final review, as they were not published articles. We included randomized controlled trials (RCTs) involving AA adult women, ages 18–64, at risk of MetS. The comparison group was no intervention or minimal intervention and we included trials that involved multifactorial interventions. Outcomes of interest were determined using PICOS (population, intervention, comparison intervention, outcome measures, and study design) criteria where major MetS, chronic disease, and CVD risk factors were characterized with the following inclusion criteria: blood pressure, blood lipids, type 2 diabetes, BMI, waist circumference, chronic disease, mental health, quality of life, stress, depression, physical activity, behavior, glucose, insulin, autoimmune, and inflammation.

### 3.2. Data Extraction and Quality Assessment

Two independent reviewers validated, extracted, and recorded relevant study data using predefined criteria. Risk of bias was assessed using the Cochrane classification, and the quality of all studies was independently assessed using the Cochrane scoring criteria [43] assigning high (0 points) or low (1 point) bias scores to each bias category. One point each was given for describing (1) the method(s) used to generate random allocation sequence, (2) the method(s) used to conceal allocation sequence, (3) the details regarding exclusions from analysis, withdrawals, and dropouts from trials, (4) measures used to blind the outcome evaluators, and (5) other biases—including reporting bias or any important concerns not addressed in other domains in the assessment tool. Using this method, a maximum of five points was awarded. Given the nature of the interventions, blinding of the study participants is not possible and was not included in the scoring of the trials. Any discrepancies in the scoring of the trials were resolved by discussion between the two reviewers.

## 4. Results

### 4.1. Study Description

**Figure 1:**
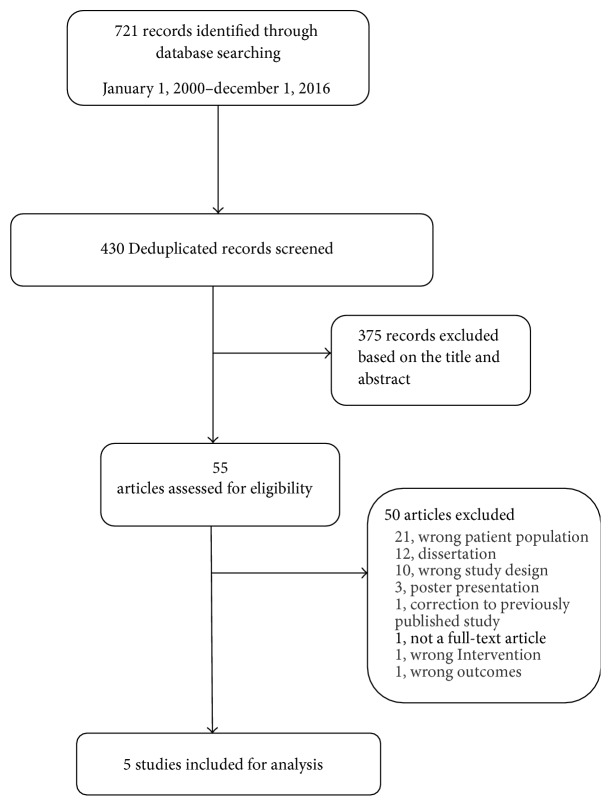
Diagram of review process and trial selection.

The database searches identified 721 potentially relevant articles, of which 430 remained after deduplication (exclusion of duplicated articles). Further, 425 were screened, assessed for full-text eligibility, and excluded resulting in five (5) trials being included in this paper. A schematic of the excluded studies as well as the reasons for exclusion is outlined in Figure 1. Reasons for exclusion for most studies included wrong patient population, dissertation, wrong study design, and poster presentation/abstract. Nine (9) studies were excluded because the intervention did not include a majority of AA women. Four (4) studies were excluded because the mean ages of the participants were unknown. Five (5) studies were excluded because they were of a single-group, quasi-experimental design. Details regarding study design, main measures, and outcomes are included in Table 1.

*Study Quality*. Details regarding bias judgment for the five included trials are presented in Table 2. The quality scores of the included RCTs ranged between 0 and 4 out of possible 5 points.

*Allocation*. The methods of random sequence generation and methods of allocation concealment were stated and judged to be at a low risk of bias in 2 of the included studies [31, 32]. In the remaining 3 studies, the method of random sequence generation and methods of allocation concealment were judged to be at high risk of bias [33–35]. Of the five included trials, only 2 adequately described the methods of randomization and reported sufficient information regarding appropriate allocation concealment [31, 32].

*Blinding*. With regard to blinding, risk of bias was high for all 5 studies. It is difficult, if not impossible, to blind participants and personnel to behavioral interventions. Therefore, the blinding of outcome assessors was excluded from scoring as high or low risk of bias.

*Incomplete Outcome Data*. In one of the included studies, we judged the reporting of incomplete outcome data to be at an unclear risk of bias [35]. This was because there was insufficient information to judge. The researchers provided no outcome data on certain variables of interest (BMI, blood pressure, etc.) due to loss to follow-up, dropouts or because the number lost to follow-up was not reported. Two studies were judged to be at high risk of bias [33, 34]. We judged the remaining 2 studies [31, 32] to be at low risk of bias as information on dropouts and numbers lost to follow-up was provided.

*Selective Reporting*. For two included studies, we judged the risk of bias for selective reporting as low since these studies [31, 34] clearly stated their outcomes and reported their results. One study [35] was judged as unclear because certain data were not presented due to loss to follow-up. Another study [32] was judged as high risk of bias because there was insufficient data to determine the methods and dosages used.

*Other Potential Sources of Bias*. For all included studies, there was insufficient information to judge the risk of bias from other potential sources.

## 5. Effects of Interventions

*Clinical Events*. None of the included studies reported type 2 diabetes diagnoses or depressive symptoms.

### 5.1. Metabolic Syndrome Risk Factors

*Blood Pressure*. Three of the five included studies provided information on blood pressure after the trial was completed [31, 33, 34]. Two studies did not report usable blood pressure data for meta-analysis [32, 35].

*Blood Lipids-Cholesterol*. Two of the included studies provided information on serum cholesterol [31, 35]. One study included information on triglycerides [31].

*Type 2 Diabetes*. None of the included studies provided information on type 2 diabetes.

*Prediabetes Outcomes: Glucose, Insulin, and HbA1c*. One of the included studies provided information on glucose, insulin, and HbA1c [31].

*Weight Change/BMI*. Two of the included studies provided information on BMI [31] and weight change [32].

*Waist Circumference*. One of the included studies provided information on waist circumference [31].

*Chronic Disease*. None of the included studies provided information on chronic disease outcomes.

*Mental Health Outcomes: Quality of Life*. Two of the included studies provided information on quality of life [31, 35].

*Mental Health Outcomes: Stress*. Four of the included studies provided information on stress [31–34].

*Mental Health Outcomes: Depression*. None of the included studies provided information on depression or depressive symptoms.

*Behavioral Outcomes: Physical Activity*. Three of the included studies provided information on physical activity [31, 32, 35].

*Autoimmune and Inflammation Outcomes: hsCRP*. One of the included studies provided information on autoimmune and inflammation outcomes measuring high sensitivity C-reactive protein [31].

### 5.2. Included Studies

Details of studies included in this review are given in Table 1. Five trials with 378 participants randomized met the inclusion criteria. The health status of participants varied between the five studies: one study recruited obese women with prediabetes [31], one study recruited women with essential hypertension [33], and one study recruited women with high stress levels [32], while the two remaining studies recruited women but did not mention their health status [34, 35]. All studies were conducted in the USA. In two trials, the intervention was not a mind-body therapy; however, the comparison group made use of a mind-body therapy included in our search terms [33, 35]. Another two studies evaluated lifestyle/behavioral modification programs augmented with mind-body therapies [31, 32], while only one intervention specifically examined the efficacy of a mind-body therapy in AA women [34].

Bernstein and colleagues (modified score = 4) conducted an RCT of a lifestyle modification program using a two-group, parallel design in overweight and obese AA women with prediabetes. Subjects (*N* = 27) were randomized to a lifestyle modification program (*n* = 14) or usual care (*n* = 13) for 6 weeks. The FRESH (Fitness, Relaxation, and Eating to Stay Healthy) program consisted of 6 weekly 90-minute sessions. The intervention primarily included health education classes with a registered dietitian leading discussions involving making healthy food choices and a chef demonstrating cooking techniques. Meetings also incorporated education about psychological stress with demonstrations of meditation and other relaxation techniques to facilitate behavioral change. Primary outcome measures were weight change and study program adherence. Other measures taken before and after the study included the following: BMI; hemoglobin A1c (HbA1c); waist circumference; fasting blood glucose; fasting insulin and insulin resistance using the homeostasis model assessment (HOMA); blood pressure; high sensitivity C-reactive protein (hs-CRP); serum cholesterol; sleep; stress as measured by Cohen's* Perceived Stress Scale*; physical activity as measured by the* Recent Physical Activity Questionnaire*; quality of life measured by* RAND-SF* and dietary habits measured by the* National Institutes of Health/National Cancer Institute ASA24 Automated Self-Administered 24-hour recall*. There were no significant differences observed in waist circumference, blood pressure, fasting glucose, hs-CRP, fasting insulin, or insulin resistance [31]. Similarly, no differences were seen in psychosocial stress, hours of sleep, physical activity, and quality of life [31]. Overall class attendance was 92%. Full attendance at the lifestyle sessions indicated an interest in relaxation, meditation, and guided imagery. No treatment effects were seen on weight (0.3 kg; 95% CI: −1.4 to 2.0 kg, *p* = 0.72), BMI (0.1 units; 95% CI: −0.5 to 0.8 units, *p* = 0.67), or HgbA1c (0.0%; 95% CI: −0.1 to 0.1%, *p* = 0.61). There were no significant differences observed in waist circumference, blood pressure, fasting glucose, hs-CRP, fasting insulin, or insulin resistance. Similarly, no differences were seen in psychosocial stress, hours of sleep, physical activity, quality of life, and perceived stress [31]. In both study arms, psychosocial stress scores were high while quality of life scores were also reported to be generally above average. Hours of sleep and physical activity were low in both groups before and after the intervention.

Jefferson (modified score = 1) evaluated the efficacy of a therapeutic chair massage intervention versus diaphragmatic breathing as an attention control in AA women with hypertension in a randomized study over the course of 6 weeks (*N* = 68). This two-group design further divided the intervention and control groups into four groups—two subgroups received therapeutic chair massage (groups 1 and 3) and two groups received information on diaphragmatic breathing (groups 2 and 4). Outcome measures included arterial blood pressure using electronic and manual cuffs, anxiety using the* State Trait Anxiety Inventory (STAI)*, and perceived stress using Cohen's* Perceived Stress Scale*. Outcomes were measured at 1-week and 6-week intervals. Significant improvements in systolic and diastolic blood pressure were observed in the chair massage intervention subgroup 3 (*p* = 0.0001 and *p* = 0.02, resp.) [33] from baseline to the 1-week measurement interval compared to those of the attention control breathing group, subgroup 4 (*p* = 0.79 and *p* = 0.26). There were no significant differences in perceived stress scores between the two groups or within the four study subgroups [33]. State Trait Anxiety Inventory scores were significantly reduced in the massage intervention subgroup 3 (*p* = 0.001) one week following the intervention compared to the attention control diaphragmatic breathing subgroup 4 (*p* = 0.09) [33].

Cox and colleagues (modified score = 3) examined the effects of a lifestyle modification intervention—Diabetes Prevention Program (DPP) Lifestyle Balance intervention using a randomized, controlled, design. The standard DPP lifestyle intervention was augmented with stress management strategies including relaxation techniques—progressive muscle relaxation, diaphragmatic breathing, and mindfulness. AA female subjects (*n* = 44) with elevated stress scores were randomized to either the DPP Lifestyle + Stress intervention group or the standard DPP Lifestyle group as a control group. Both groups were offered 12 weekly group sessions that lasted 60 minutes and were delivered by AA counselors trained in health behavior/health promotion. Self-monitoring diaries were provided weekly to record dietary intake and physical activity. Outcome measures included adherence using class/session attendance and self-monitoring diaries, stress levels using Cohen's* Perceived Stress Scale*, and salivary cortisol as a physiological measure of stress. There was no significant difference in overall class attendance and diary adherence patterns between the two treatment groups. Significant reductions in body weight (*p* < 0.001) were observed in both groups after intervention [32]. Stronger positive associations between group attendance and weight loss were observed in the Lifestyle + Stress group compared with the Lifestyle Alone group. Women in both groups reported lower perceived stress scores after intervention (*p* = 0.01) [32]. Weight loss was significantly associated with total sessions attended (*r* = .31, *p* = 0.04) and total self-monitoring diaries submitted (*r* = .39, *p* = 0.01) [32]. Those who lost more weight reported lower follow-up PSS (*r* = −.35, *p* = 0.03). The study demonstrates the feasibility of recruiting overweight individuals with the MetS for a yoga study. The study demonstrated the potential for the standard DPP protocol to be enhanced with stress management techniques leading to improved outcomes in high-risk AA women.

Young and Stewart (modified score = 2) evaluated the effectiveness of a 6-month aerobic exercise intervention versus a stretching and health lecture intervention in a study of 196 AA women attending churches (*N* = 11) randomized to one of the two aforementioned treatment conditions. Psychosocial factors: self-efficacy for exercise, social support for exercise, and health-related dimensions of quality of life were measured at baseline. Outcome measures included BMI, blood pressure using an automated blood pressure monitor, serum cholesterol, daily levels of energy expenditure and physical activity as measured by a modified Balke protocol, the Stanford 7-day Physical Activity Recall (PAR), and the Yale Physical Activity Survey (YPAS)

Significant differences were not seen in physical activity levels [35] (*p* = 0.03) in the aerobic exercise treatment group (*n* = 5; 123 participants) in comparison to the stretch and health comparison group (*n* = 6; 73 participants). Attendance in classes averaged 24.3% over the 6-month period with higher attendance (31.5%, *p* < 0.03) in the stretch and health comparison group. Increased levels of self-efficacy and social support [35] from family and from friends at baseline (*p* = 0.008 and *p* = 0.005, resp.) significantly predicted change in physical activity status regardless of treatment group [35].

Webb and colleagues (modified score = 1) examined the efficacy of a 7-muscle group progressive relaxation intervention versus set aside time for relaxation in AA women (*N* = 43) employed in a health service setting over the course of 8 weeks. Outcome measures included systolic, diastolic, and mean arterial blood pressure using an automated blood pressure monitor and the negative effects of the stress response using the* Personal Strain Questionnaire*. Significant improvements in interpersonal strain [34] (*F*[2, 40] = 3.28, *p* = 0.02) were observed in the experimental group. Similarly, within-subjects improvements in interpersonal strain scores (*F*[2, 40] = 18.00, *p* = 0.0001) and physical strain scores (*F*[2, 40] = 15.44, *p* = 0.001) were observed over time. No significant differences in blood pressure [34] were observed.

## 6. Discussion

The authors screened 430 studies and identified five trials that randomized 378 participants in studies of six-week to six-month duration. Only 3 of these papers provided usable data for meta-analysis. For the remaining studies, either no CIs or standard deviations for the intervention or control group were included. We identified no ongoing studies.

The included RCTs measured outcomes associated with MetS and cardiometabolic disease but they were short term. There were no significant differences between treatment groups observed for BMI, waist circumference, fasting glucose, insulin resistance, cholesterol, and hs-CRP in any of the studies. There were some favorable effects on blood pressure in two trials (the progressive muscle relaxation intervention group [34] and the chair massage intervention group) [33] as well as improvements in body weight in both intervention and control groups of another trial (in the DPP Lifestyle Balance trial) [32]. In terms of psychosocial/behavioral factors (e.g., sleep, stress, quality of life, and physical activity), there were favorable effects on interpersonal strain scores in one trial (between the progressive muscle relaxation experimental and control groups; similarly, within-group improvements were also seen over time in the PMR experimental group) [34]. Additionally, perceived stress was lowered in both groups participating in the DPP Lifestyle Balance Program trial [32] while anxiety scores lowered in the chair massage intervention subgroup [33]. None of the trials reported on our other outcomes of interest. Heterogeneity between the two trials with usable data precluded meta-analysis.

### 6.1. Overall Completeness and Applicability of Evidence

This review included adults who were at different levels of CVD risk and included AA women. All trials were published in the USA. None of the included studies reported on the sustained effects of mind-body interventions on MetS and CVD risk, but this may be because the included studies were small, with short-term follow-up. Only one of five included trials reported on the full spectrum of MetS outcomes including waist circumference, blood pressure, lipid profile, and fasting glucose [31]. Given the physiological outcomes associated with the MetS, objective outcome measures such as lipoprotein profiles, circulating levels of glucose and insulin, and anthropometric measures are consistent across studies of the MetS, which allows for comparison among studies. However, different subjective or psychosocial outcomes such as fatigue and health-related quality of life make comparisons more challenging. This issue is not unique to the study of mind-body or complementary therapies.

Indeed, RCTs often use various outcome measures of patient symptoms to quantify the same concepts, limiting comparison across studies. We were not able to examine the effects of baseline CVD risk or the duration of mind-body therapies because of the limited number of trials included. This review identified only five trials, two of which had questionable applicability, as they did not examine the efficacy of a mind-body therapy listed in our trial selection criteria [33, 35]. Only one trial examined the efficacy of a mind-body therapy alone [34]. The remaining two trials [31, 32] evaluated the effectiveness of mind-body therapies as part of multiple risk factor lifestyle interventions. These factors reduced our confidence in the limited results available to date. We could not rigorously assess mind-body therapies since the few included trials were relatively short term, being of only 6-week to 6-month duration. As a result, it is unclear if any of the effects of mind-body therapy can be sustained in the long term. For the results of a clinical study to be useful, one must be able to replicate the trial; therefore, all aspects of the methodology and the intervention, as well as a detailed description of the results, must be reported. None of the included studies in this paper provided a clear rationale for the treatment specificity or duration. Given that the optimal dosage of mind-body therapies has yet to be determined, a description of the treatment duration and number of treatments should be included. Furthermore, there was a considerable heterogeneity between trials for blood pressure meaning that the findings for this outcome can only be suggestive. Though the studies support the potential clinical effectiveness of mind-body practices in improving indices of the MetS, more studies are required given the included trials had several limitations.

### 6.2. Quality of the Evidence

The results of this review should be treated with caution since the included trials were at a high risk of bias. In 3 of the included studies, the methods of the random sequence generation were not stated or unclear. In the same 3 trials, the details of allocation concealment were not provided. None of the included studies reported that the outcome assessors were blind. However, it is difficult, if not impossible, to blind participants and personnel to behavioral interventions such as mind-body therapies. While there remains a lack of rigorous trials that apply adequate methodology, including the use of blinding and placebo treatments, given that trials with inadequate levels of blinding are likely to show exaggerated treatment effects, the nature of mind-body therapies makes it seemingly impossible to blind subjects to the intervention or to develop a placebo.

We judged risk of bias related to incomplete outcome data as low in 3 of the 5 included studies. For all studies, we judged the risk of other biases as unclear as there was insufficient information to judge. This review was also at risk of small-study bias since the included studies were relatively small. Limitations of systematic reviews, including the current paper, relate to any potential incompleteness of the reviewed studies. This effect may result from publication bias given that negative studies tend to remain unpublished. In addition, we were unable to examine the effects of publication bias in funnel plots because of the limited number of trials included. Nonetheless, small trials are often carried out with less rigor methodologically speaking, are more likely to be conducted in selected populations, and have been shown to report larger beneficial effects than larger trials [44–46].

### 6.3. Potential Biases in the Review Process

In this review, we conducted a comprehensive search across major databases for interventions involving mind-body therapies for AA female populations. Two review authors independently performed screening, inclusion and exclusion, data abstraction, data entry, and analysis. Our decision to include trials that involved mind-body therapies in combination with other behavioral interventions introduced the possibility for potential confounding effects of other behavioral approaches on our outcomes. This was done to expand the number of trials eligible for inclusion. The inclusion of studies focusing on clinical outcomes related to single components of the MetS may be a limitation; however, the inclusion of trials examining only certain cardiometabolic measures would not provide reliable data on the clinical effectiveness of mind-body therapies in improving the symptom cluster that comprises the MetS as a whole. In addition, the small number of trials on which this review was based, limitations in the reporting of methodology, a high risk of bias in most studies, and sparse or no data for our outcomes mean that the findings of this review are currently extremely limited.

### 6.4. Implications for Practice and Research

Only five trials met the inclusion criteria for our review and only one reported our primary outcomes. There was considerable heterogeneity between the included trials for outcomes including blood pressure meaning that meta-analysis was not possible. Therefore, any findings with regard to this outcome can only be suggestive. None of the studies indicated the use of ambulatory blood pressure monitoring over the course of the study. Measurement of blood pressure only at study intervals might have underestimated the efficacy of the mind-body interventions in lowering this key component/symptom of the MetS. None of the included studies reported on triglycerides, occurrence of type 2 diabetes, or depressive symptoms. The trials in this review were also at overall serious risk of bias and, as such, results should be treated with caution. Mind-body therapies may carry practical advantages as therapeutic interventions for managing the symptom cluster associated with the MetS. However, there are currently few randomized controlled trials that meet our inclusion criteria to examine the effects of mind-body therapies for the prevention and management of MetS. At present, there is a shortage of large, long-term trials on the effectiveness of mind-body therapies for the primary prevention of MetS disease in AA women at risk for cardiometabolic diseases. Furthermore, we found only one trial that measured MetS-associated outcomes in AA women. As such, high-quality, large trials with long-term follow-up that measures a broader range of outcomes are needed in order to determine the effectiveness of mind-body therapies.

## 7. Conclusion

There is a need to identify cost-effective prevention and management strategies for the MetS that address the multiple interrelated factors underlying this complex and high-risk symptom cluster. No such research has been conducted in this regard with respect to mind-body therapies in this vulnerable population. Current clinical practice guidelines indicate lifestyle modifications as the recommended therapy for prehypertension, as well as other indicators of the MetS. Given the positive effects of mind-body therapies on cardiometabolic components, these modalities most likely would be of benefit to individuals with MetS. The current paper provides healthcare practitioners with information that could be used in decision-making about recommendations involving mind-body practices. In light of the important role of psychosocial factors in the development of insulin resistance, T2D, and other chronic diseases, the influence of sympathetic activation in the pathogenesis of insulin resistant states and the bidirectional relationships of these and other insulin resistance related risk factors and mind-body therapies may hold promise for both the prevention and treatment of the MetS. Because RCTs remain the “gold standard” in biomedical research, this paper highlights the need for such trials of mind-body therapies with regard to the management of the MetS, given the relative absence of such studies in the literature, as well as the mechanisms of action involved in mind-body therapies.

## Figures and Tables

**Table 1:** Characteristics of included studies.

References	Design	Mean age, sample size, Metabolic syndrome-related condition	Intervention-treatment regimen	Main outcome measures	Main results	Comments
Bernstein et al. 2014 [31]	RCT	Mean age = 56 Sample size = 27 Prediabetes	Low-intensity exercise; mindfulness; relaxation techniques: meditation and guided imagery	Weight/BMI; HbA1c; waist circumference, blood pressure, CRP, fasting insulin; PSS, physical activity	No significant treatment effect of the lifestyle intervention on weight (0.3 kg; 95% CI: −1.4 to 2.0 kg, *p* = 0.72), BMI (0.1 units; 95% CI: −0.5 to 0.8 units, *p* = 0.67), or HbA1c (0.0%; 95% CI: −0.1 to 0.1%, *p* = 0.61) or other measures	92% class attendance; trends towards improvement in healthy eating and cooking habits were seen

Cox et al. 2013 [32]	RCT	Mean age = 44.5 Sample size = 44 Obesity/moderate-to-high stress levels	Diabetes prevention program + guided relaxation; diaphragmatic breathing; mindfulness	Weight loss; stress-PSS; stress-salivary cortisol	Stronger positive association between group attendance and weight loss in the treatment group (*p* = 0.01); trend towards greater reductions in salivary cortisol in the treatment group (*p* = 0.20); no difference in PSS	86% retention rates; weight loss associated with total sessions attended (*r* = .31, *p* = 0.04) and total self-monitoring diaries submitted (*r* = .39, *p* = 0.01)

Jefferson 2010 [33]	RCT	Mean age = 52.9 Sample size = 68 Hypertension	Therapeutic chair massage; patient-taught diaphragmatic breathing	Blood pressure; anxiety-STAI; stress-PSS	Significant differences in systolic blood pressure (*p* = 0.01), diastolic blood pressure (*p* = 0.02), STAI (*p* = 0.01)	No significant differences in PSS between the massage groups (6-week, *p* = −0.43; 1-week, *p* = 0.17) and breathing groups 6-week, *p* = 0.29; 1-week, *p* = 0.19)

Webb et al. 2000 [34]	RCT	Mean age = 33.5 Sample size = 43 Hypertension	Seven-muscle group progressive relaxation	Blood pressure; Physical, Interpersonal, Psychological Strain scores	Within-subjects changes over time in interpersonal strain scores (*F*[2, 40] = 18.00, *p* = 0.0001) and physical strain scores (*F*[2,40] = 15.44, *p* = 0.001); psychological strain within subjects (*F*[2, 40] = 12.20, *p* = 0.0001)	Between-groups interactions greater reductions in interpersonal strain (*F*[2, 40] = 3.28, *p* = 0.02) and physical strain (*F*[2, 40] = 4.98, *p* = 0.01).

Young and Stewart 2006 [35]	Cluster RCT	Mean age = 48.3 Sample size = 196 Physical inactivity	Alternating weekly low-intensity stretching classes and health education	Blood pressure; cholesterol-HDL-C	Exercise participants attended an average of 21.6% of classes, whereas the stretch and health participants attended an average of 31.5% of classes (*p* < 0.03).	Participants who did not return for follow-up were younger than those who did return (47.3 ± 8.7 versus 51.1 ± 9.6, *p* < 0.009).

RCT: randomized controlled trial; PSS: Cohen's Perceived Stress Scale; STAI: State Trait Anxiety Inventory; HDL-C: high density lipoprotein-cholesterol.

**Table 2:** Risk of bias summary.

Author, year	Title of trial	Reference number	Modified bias score	Random sequence generation	Allocation concealment	Incomplete outcome data	Selective reporting	Blinding of outcome assessment
Bernstein et al. 2014	Management of Pre-Diabetes through Lifestyle Modification in Overweight and Obese African-American Women: The Fitness, Relaxation, and Eating to Stay Healthy (FRESH) Randomized Controlled Trial	[31]	4	Low	Low	Low	Low	Not judged

Cox et al. 2013	Stress Management-Augmented Behavioral Weight Loss Intervention for African American Women: A Pilot, Randomized Controlled Trial	[32]	3	Low	Low	Low	Unclear	Not judged

Jefferson 2010	Exploring Effects of Therapeutic Massage and Patient Teaching in the Practice of Diaphragmatic Breathing on Blood Pressure, Stress, and Anxiety in Hypertensive African-American Women: An Intervention Study	[33]	1	High	High	High	Low	Not judged

Webb et al. 2000	A Progressive Relaxation Intervention at the Worksite for African-American Women	[34]	1	High	High	High	Low	Not judged

Young and Stewart 2006	A Church-Based Physical Activity Intervention for African American Women	[35]	2	High	High	Unclear	Unclear	Not judged
